# Supplementary material for: Non-Human Primate-Derived Adenoviruses for Future Use as Oncolytic Agents?
Source: Int J Mol Sci. 2020 Jul 8;21(14):4821. doi: 10.3390/ijms21144821 (PMC7404033; doi:10.3390/ijms21144821)
Supplement: Supplementary file 1 [file ijms-21-04821-s001.pdf]

Supplementary Material

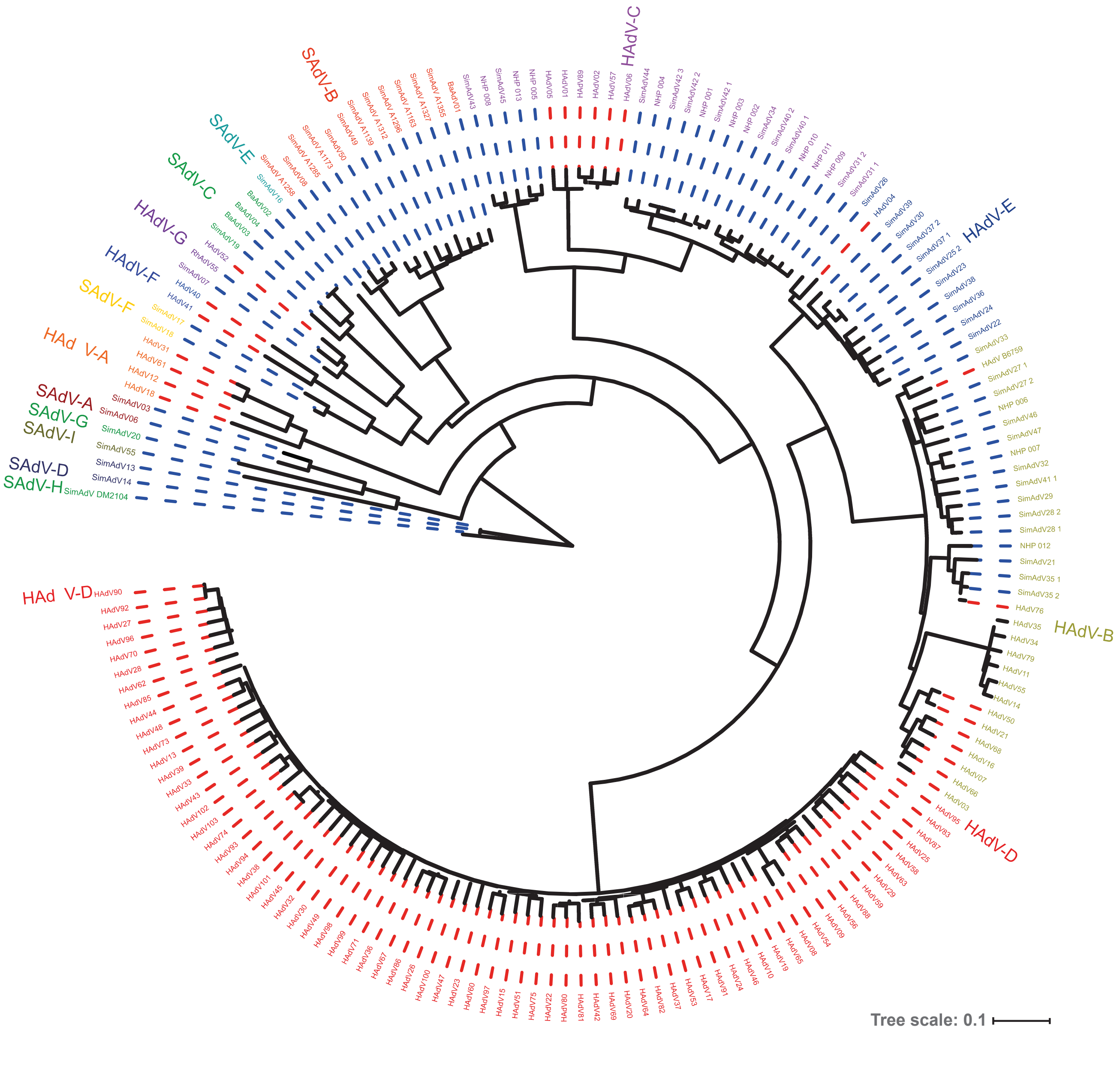

Figure S1. A high-resolution phylogenetic tree representing the genetic resemblance of human- and non-human primate-derived adenoviruses.
